# Efficacy and safety of dapagliflozin in the treatment of chronic heart failure: A protocol for systematic review and meta-analysis

**DOI:** 10.1097/MD.0000000000026420

**Published:** 2021-07-02

**Authors:** Xueyan Dong, Lili Ren, Yueli Liu, Xuewei Yin, Siyuan Cui, Wulin Gao, Liming Yu

**Affiliations:** a Affiliated Hospital of Shandong University of Traditional Chinese Medicine; b First College of Clinical Medicine, Shandong University of Traditional Chinese Medicine; c The Third Affiliated Hospital of Shandong First Medical University, Jinan, Shandong Province, China.

**Keywords:** chronic heart failure, dapagliflozin, meta-analysis, systematic review

## Abstract

**Background:**

As the last link in the chain of cardiovascular events, chronic heart failure (CHF) has high morbidity, high mortality, and poor prognosis. It is one of the main causes of death and disability worldwide. As a new drug for the treatment of chronic cardiovascular disease, dapagliflozin, the efficacy, and safety issues are still the focus of attention. Therefore, we conducted a meta-analysis to evaluate the efficacy and safety of dapagliflozin in the treatment of CHF.

**Methods:**

According to the search strategy, regardless of publication date or language, randomized controlled trials (RCTs) of dapagliflozin for CHF will be retrieved from 8 databases. First of all, the literature was screened according to the eligibility criteria, and use the Cochrane Collaboration's tool to assess the quality of the included literature. Then, using Rev Man 5.3 and STATA 14.2 software for traditional meta-analysis. Finally, the evaluation of the quality of the evidence and the strength of the recommendations will adopt the Grading of Recommendations, Assessment, Development and Evaluation method.

**Results:**

This study will evaluate the efficacy and safety of dapagliflozin for CHF, thereby providing more evidence support for clinical decision-making in CHF.

**Conclusion:**

Our research will provide more references for the clinical medication of patients with CHF.

**Protocol registration number:**

INPLASY202150046

## 1 Introduction

Chronic heart failure (CHF) is a group of complex clinical syndromes caused by abnormal changes in cardiac structure and (or) function caused by various reasons, which make ventricular contraction and (or) diastolic dysfunction occur.^[1]^ The main manifestations are dyspnea, fatigue, and fluid retention, etc. CHF is the end-stage manifestation of cardiovascular disease and the main cause of death. It has a high recurrence rate and a poor prognosis, which seriously threatens people's physical and mental health.^[2]^ In developed countries, the prevalence of HF in adults is about 1% to 2%, and it gradually increases with age, and exceeds 10% in people over 80.^[2,3]^ After being diagnosed with CHF, the rehospitalization rate was as high as 83.1%, and 42.6% were hospitalized >4 times.^[4]^ The Framingham Heart Study in the United States shows that within 5 years of the initial diagnosis of CHF, the mortality rate of patients is about 50%.^[5]^ In patients with acute myocardial infarction with CHF, the 1-year mortality rate is >50%.^[3]^ Therefore, extending the survival period of patients with CHF, reducing the mortality rate, and improving the prognosis are key issues that need to be resolved.

Dapagliflozin is a new type of hypoglycemic agent belonging to the sodium-glucose transporter 2 inhibitors (SGLT-2i) class.^[6]^ Its effect of lowering blood sugar does not depend on improving insulin secretion and peripheral tissue resistance to insulin. It mainly inhibits the activity of proximal renal tubules SGLT-2, reduces the reabsorption of glucose by the renal tubules, thereby increasing the excretion of glucose in the urine, lower blood sugar. In addition, SGLT-2i drugs also have unique effects other than hypoglycemic effects. For example, empagliflozin and canagliflozin have been proven to have additional cardiovascular protection and can reduce the occurrence of cardiovascular events.^[7,8]^ As a similar drug, dapagliflozin has also been proven to benefit cardiovascular disease in some studies, and this effect is independent of the hypoglycemic effect. In dapagliflozin and prevention of adverse outcomes in heart failure trial, dapagliflozin can reduce hospitalization rates and mortality from cardiovascular events in patients with and without type 2 diabetes mellitus (T2DM) who had HF with reduced ejection fraction (EF).^[9]^ However, some studies found that dapagliflozin can reduce the hospitalization rate of patients with CHF in the analysis of the baseline data and EF value of the DECLARE-TIMI 58 trial, regardless of whether the ejection fraction is normal or not.^[10]^ In addition, people may worry about the potential safety issues of this new drug in the treatment of CHF.^[11]^ Therefore, this study proposes a systematic review program to evaluate the efficacy and safety of dapagliflozin on CHF, and to provide sufficient basis for further guidance of clinical medication, so as to avoid unnecessary traps.

## 2 Methods

### 2.1 Research registration

Our protocol has been registered on the International Platform of Registered Systematic Review and Meta-Analysis Protocols (INPLASY). The number was INPLASY202150046 (URL=https://inplasy.com/inplasy-2021–5–0046/). We will be based on the Preferred Reporting Items for Systematic Review and Meta-Analysis Protocols (PRISMA-P), and strictly follow the requirements and conduct.^[12]^

### 2.2 Data sources and retrieval strategy

We will conduct a literature search from the following electronic databases: PubMed, EMBASE, the Cochrane Library, Web of Science, CNKI, Wan-fang Data, Chinses Biomedical Literature Database, Chinese Scientific Journal Database. There are no restrictions on publication date and language. In addition, the references listed in each included article are also manually searched.

Retrieve the databases by combining subject words with random words. Appropriate adjustments will be made according to the grammatical rules of different databases to ensure the completeness and comprehensiveness of the search. We will first conduct a pre-search, and discuss the problems encountered in the search process with the team. After confirming that there are no problems, we will conduct a formal literature search. Taking PubMed as an example, the retrieval strategy is shown in Table 1.

**Table 1:** Search strategy for PubMed database.

Number	Search item
#1	Heart Failure[Mesh]
#2	Heart Failure[Title/Abstract] OR Cardiac Failure[Title/Abstract] OR Heart Decompensation[Title/Abstract] OR Decompensation, Heart[Title/Abstract] OR Heart Failure, Right-Sided[Title/Abstract] OR Heart Failure, Right Sided[Title/Abstract] OR Right-Sided Heart Failure[Title/Abstract] OR Right Sided Heart Failure[Title/Abstract] OR Myocardial Failure[Title/Abstract] OR Congestive Heart Failure[Title/Abstract] OR Heart Failure, Congestive [Title/Abstract] OR Heart Failure, Left-Sided[Title/Abstract] OR Heart Failure, Left Sided[Title/Abstract] OR Left-Sided Heart Failure[Title/Abstract] OR Left Sided Heart Failure[Title/Abstract]
#3	#1 OR #2
#4	Dapagliflozin[Mesh]
#5	Dapagliflozin[Title/Abstract] OR Farxiga[Title/Abstract] OR Forxiga [Title/Abstract]
#6	#4 OR #5
#7	randomized controlled trial[Title/Abstract] OR controlled clinical trial[Title/Abstract] OR RCT[Title/Abstract] OR randomized[Title/Abstract] OR randomly[Title/Abstract]
#8	#3 AND #6 AND #7

### 2.3 Eligibility criteria

We will formulate the inclusion and exclusion criteria for this study based on the PICOS principles.

#### 2.3.1 Participants

Patients with CHF, whether diagnosed by a clinician, or by any recognized criteria diagnosis of CHF, will be included. There are no restrictions on nationality, age, sex, or race. Patients who have received acute heart failure, or patients with severe liver and kidney, or blood diseases, or malignant tumors, or other uncontrolled systemic diseases are excluded.

#### 2.3.2 Interventions and comparators

The treatment group was given dapagliflozin (5–10 mg) on the basis of routine western medicine of CHF. The control group was only given routine western medicine, or the same dose of placebo was given on the basis of routine western medicine. Routine western medicine mainly includes diuretics, ACEI, ARB, β-receptor blockers, ivabradine, digitalis and inotropic drugs, vasodilators, anticoagulants, etc.^[1]^

#### 2.3.3 Outcomes

The primary outcomes include mortality and HF rehospitalization rate; the secondary outcomes include New York Heart Association classification (NYHA classification), EF, N terminal pro B type natriuretic peptide (NT-proBNP), quality of life (QOL), etc; the safety indicators include hypovolemia, hypoglycemia, kidney damage, infections of the genitourinary system, and other adverse reactions.

#### 2.3.4 Type of studies

Randomized controlled trials (RCTs) will be included in this study irrespective of language or publication category. Animal trials, review article and studies with incorrect RCT designs will be excluded.

### 2.4 Literature screening and data extraction

**Figure 1:**
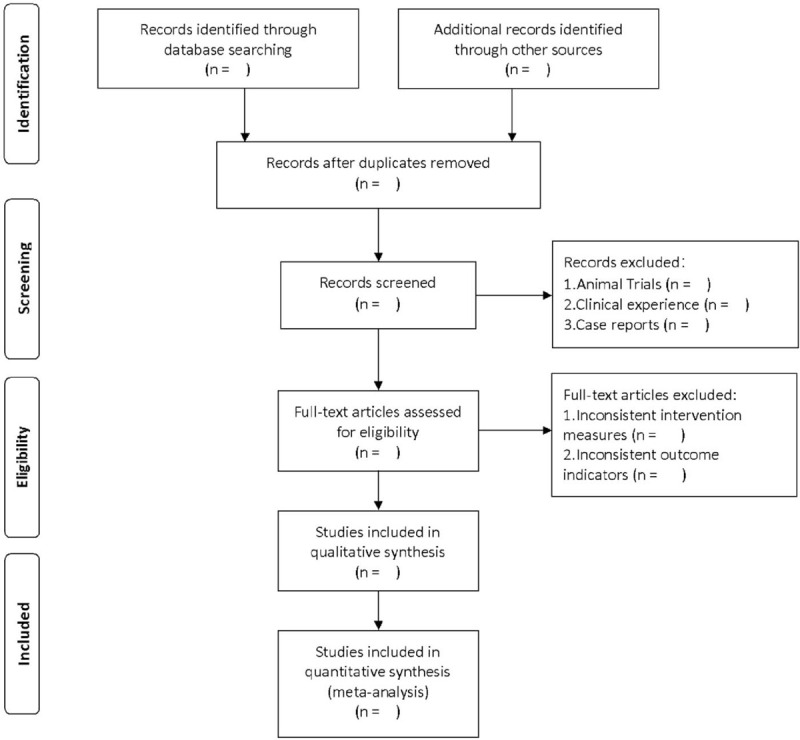
Flow diagram of literature retrieval.

Use endnoteX9.0 software to manage literature. After searching literature based on the above steps, import them into endnote software for literature screening. First, 2 independent researchers will conduct a preliminary literature screening based on the titles and abstracts of the included literature to eliminate duplicate and non-RCTs. Then read the full text of the remaining literature according to the previously designed principles of eligibility criteria, and finally determine the appropriate literature. When 2 researchers disagree, a third researcher will resolve it. The specific screening process is shown in Fig. 1.

According to the Cochrane Handbook for Systematic Reviews of Interventions, 2 researchers independently extracted and recorded the required information from all the included literature. When 2 researchers disagree, they will discuss to reach an agreement, otherwise they will work with the third researcher to resolve. The required information mainly includes the author, publication time, study design, participants number and demographic characteristics (age, sex, etc), treatment status (e.g., the initial dose and treatment period of dapagliflozin), and outcomes (e.g., mortality, heart failure rehospitalization rate). If any of the above information in the included literature is incomplete, we will contact the corresponding author via email to obtain the required data.

### 2.5 Quality assessment

Two reviewers will independently assess the quality of the included literature according to the Cochrane Collaboration's tool for RCTs. If there is a disagreement between 2 reviewers, the third reviewer resolves the issue. According to Cochrane Handbook V.5.2.0, characteristics of each item will be evaluated in 3 categories: low, unclear, and high.^[13]^ The results of the quality assessment will be completed using software Review Manager 5.3.

### 2.6 Statistical analysis

#### 2.6.1 Traditional meta-analysis

We will execute Rev Man 5.3 and STATA 14.2 software for traditional meta-analysis. For dichotomous data, we will calculate a summary estimate with 95% confidence interval odds ratio value; for continuous data, we will calculate a summary estimate of standardized mean difference with 95% confidence interval, and *P* < .05 is considered statistically significant. The heterogeneity among the included literature will be assessed using the *Q* test method and *I*^2^ statistic method. When the *Q* statistic corresponds to *P* ≤ .10 or *I*^2^ > 50%, it indicates that there is heterogeneity among the included literature, and assess the effect size by the random effect; on the contrary, a fixed effect model is used.

#### 2.6.2 Subgroup analysis

Taking into account the issue of heterogeneity, we will conduct a subgroup analysis based on the specific circumstances of the included literature. If there is a problem of heterogeneity, we will conduct a subgroup analysis of age, sex, and interventions. In addition, in order to understand whether the left ventricular ejection fraction (LVEF) will affect the efficacy of dapagliflozin, it will be used for CHF patients with LVEF <40% and CHF with unknown LVEF the mortality rate and rehospitalization rate are analyzed by subgroups. In order to understand whether patients with CHF and T2DM affect the efficacy of dapagliflozin, the mortality rate and rehospitalization rate will be subgroups-analyzed on whether patients with CHF have T2DM.

#### 2.6.3 Sensitivity analysis

This systematic review will use the method of eliminating each study one by one for sensitivity analysis. If the effective indicators (e.g., the mortality rate and rehospitalization rate) of dapagliflozin in the treatment of CHF have not changed significantly, it indicates that the study is robustness. On the contrary, it is not robustness. According to the specific situation, low-quality research is excluded.

#### 2.6.4 Publication biases

Publication biases will be assessed by a funnel plot for meta-analysis and quantified by the Egger method. It should be noted that if the number of included literature is ≥10, it is appropriate to use a funnel plot to assess potential publication bias. However, if the included literature is <10, it may affect the overall test power because the included number is too small, and it is difficult to accurately evaluate the symmetry of the funnel plot.

### 2.7 Evidence quality assessment

The Grading of Recommendations, Assessment, Development and Evaluation used to assess the quality of evidence. The quality of evidence is divided into 4 levels from 5 aspects, namely high, medium, low, and very low.^[14]^

### 2.8 Ethical considerations and dissemination plans

This study is a systematic review, it does not involve medical ethics and patients’ informed consent. This study will publish the results of meta-analysis in journal papers.

## 3 Discussion

As the aging of the population in various countries gradually accelerates, the incidence of various cardiovascular-related diseases is gradually increasing, such as coronary heart disease, hypertension, dyslipidemia, diabetes, Obesity, etc, and most of the end stages of these diseases end in CHF. However, in the current limited medical technology, the cardiac function and structure cannot be completely reversed temporarily. Some drugs and methods can only be used to improve or delay the deterioration of cardiac function, prevent further cardiac remodeling, reduce cardiovascular death, and improve long-term prognosis. With the discovery of neuroendocrine mechanism, early and comprehensive intervention of cardiovascular events can improve the prognosis, conventional treatments are mostly neuroendocrine antagonists, such as ACEI/ARB, β-receptor blockers, and digoxin, etc. Nevertheless, the mortality and rehospitalization rate of patients with CHF remains high. How to develop new drugs and therapies on the basis of evidence-based medicine to improve the prognosis of patients with CHF has become one of the health problems concerned by medical researchers all over the world.

Dapagliflozin is a new drug for cardiovascular diseases. Although the mechanism of SGLT-2i drugs for cardioprotection is still controversial, it may be that well-controlled blood sugar reduces the cardiotoxicity of blood sugar, or osmotic diuresis reduces water and sodium retention, thereby reducing blood volume, reducing cardiac preload, or lowering blood pressure and relieving arteriosclerosis, reducing the afterload of the heart, or improving the structure of damaged myocardial cells by adjusting oxidative stress, ion concentration and energy metabolism levels, delaying myocardial remodeling, and improving long-term prognosis.^[15–18]^ But regardless of the mechanism of action, from the existing data, dapagliflozin is beneficial to improve the prognosis of CHF.^[19,20]^ Perhaps it can bring hope to the treatment of CHF.

Although this systematic review has many advantages, such as statistical power and accuracy of effect size estimation, subgroup analysis of factors affecting treatment effects, a more objective evaluation of evidence and a more accurate and objective evaluation of effect indicators evaluation. However, it also has some problems. For example, this study is an evaluation of published literature, and there may be problems such as unscientific and non-strict RCT design, resulting in uneven quality of literature research, which affects the credibility of this study. Or the research results included in the literature have false negatives and false positives. In any case, this systematic review can provide reliable results for CHF, and provide strong evidence for the significant advantages of dapagliflozin in the treatment of CHF.

## Author contributions

**Conceptualization:** Xueyan Dong, Liming Yu.

**Data curation:** Wulin Gao, Lili Ren.

**Formal analysis:** Lili Ren, Xueyan Dong.

**Funding acquisition:** Siyuan Cui, Liming Yu.

**Methodology:** Xueyan Dong, Lili Ren, Xuewei Yin.

**Project administration:** Liming Yu.

**Writing – original draft:** Xueyan Dong, Yueli Liu, Liming Yu.

**Writing – review & editing:** Xueyan Dong, Liming Yu.
